# Optimization of an Antibody Light Chain Framework Enhances Expression, Biophysical Properties and Pharmacokinetics

**DOI:** 10.3390/antib8030046

**Published:** 2019-09-06

**Authors:** Patrice Douillard, Michael Freissmuth, Gerhard Antoine, Michael Thiele, Daniel Fleischanderl, Peter Matthiessen, Dirk Voelkel, Randolf J. Kerschbaumer, Friedrich Scheiflinger, Nicolas Sabarth

**Affiliations:** 1 Baxalta Innovations GmbH (part of Takeda), Indusriestrasse 67, 1221 Vienna, Austria; 2 Institute of Pharmacology, Centre of Physiology and Pharmacology, Medical University Vienna, 1090 Vienna, Austria

**Keywords:** antibody engineering, thermal stability, antibody expression, pharmacokinetics, framework, germline, MIF, aggregation

## Abstract

Efficacy, safety, and manufacturability of therapeutic antibodies are influenced by their biopharmaceutical and biophysical properties. These properties can be optimized by library approaches or rationale protein design. Here, we employed a protein engineering approach to modify the variable domain of the light chain (VL) framework of an oxidized macrophage migration inhibitory factor (oxMIF)-specific antibody. The amendment of the antibody sequence was based on homology to human germline VL genes. Three regions or positions were identified in the VL domain—L1-4, L66, L79—and mutated independently or in combination to match the closest germline V gene. None of the mutations altered oxMIF specificity or affinity, but some variants improved thermal stability, aggregation propensity, and resulted in up to five-fold higher expression. Importantly, the improved biopharmaceutical properties translated into a superior pharmacokinetic profile of the antibody. Thus, optimization of the V domain framework can ameliorate the biophysical qualities of a therapeutic antibody candidate, and as result its manufacturability, and also has the potential to improve pharmacokinetics.

## 1. Introduction

Therapeutic antibodies are the most important biopharmaceuticals, and their share in the market of all licensed drugs is continuously on the rise [1]. In order to fulfil the promise of new drugs, therapeutic antibodies have to be safe and efficacious. Moreover, the production of drugs needs to comply with the increasing demand for successful therapies, and costs of goods have to be minimized. Antibody yields can be increased by improving vector systems and expression systems, cell engineering, ameliorating upstream and downstream processes [2], but also by directly engineering the respective antibody. Yield, the safety and efficacy of antibodies are linked to biophysical properties such as solubility, stability, and aggregation propensity. Stability and aggregation are important factors since they impact immunogenicity, in vivo half live, dosing route, shelf life, protein production, and formulation [3]. Aggregation may result in the formation of anti-drug antibodies and drug immune complexes [4,5,6]. This can elicit adverse effects like infusion reactions, cytokine release syndrome, and anaphylaxis. In addition, the propensity to form aggregates also influences the pharmacokinetic properties of the drug by decreasing the half-life. The resulting lower drug exposure translates into reduced efficacy in vivo [7]. Aggregation is also linked to the thermal stability of a protein because an unstable protein is more susceptible to (partial) denaturation, which promotes aggregation [8,9,10]. Thermal stability affects the expression and thereby protein production [11,12]. Chemical degradations such as oxidation, isomerization, or deamidation can decrease target binding and therefore potency if the complementarity-determining region (CDR) of the antibody is involved [8].

The biopharmaceutical properties of a therapeutic antibody can be optimized during formulation development by adjusting the buffer system and the pH, by including additives etc. However, stability and aggregation can be addressed during lead antibody optimization. This facilitates later development by enhancing the developability of the molecule. Stability and aggregation propensity have been improved by sequence- and structure-based approaches [3,13,14]. However, a reliable set of empirical rules to predict the effects of mutations on protein stability is still missing [15,16]. Therefore, an experimental verification of a plethora of possible mutations in any sequence position is necessary to optimize the antibody. Antibody optimization can be achieved by protein engineering based on a library approach [17] or by a rational mutagenesis approach. The latter may aim for optimizing hydrophobic surface patches, charged residues, variable domain of the light chain/heavy chain (VH/VL) interface residues, etc. [8]. Moreover, it has been hypothesized that germline V genes have been optimized by evolution for high expression and stability [18]. Hence, in the present study, we modified a given antibody framework sequence to match it with the most homologous germline V genes in order to improve its biopharmaceutical properties. We optimized the biopharmaceutical properties of antibody BaxM159, which targets the oxidized form of macrophage migration inhibitory factor (oxMIF). OxMIF is the disease-related conformational isoform of MIF [19,20] and a promising drug target for immunological diseases and oncology [19,21]. BaxM159 was isolated from a phage-display antibody library [22], and its pharmacological efficacy was demonstrated in vitro and in vivo, in models of inflammation disease and cancer [19,20,21,22,23]. The framework optimized version(s) of the oxMIF specific antibody BaxM159 showed improved biopharmaceutical properties such as better thermal stability, aggregation resistance and expression. Moreover, the optimized version had a superior pharmacokinetic profile compared to the parental antibody.

## 2. Materials and Methods

### 2.1. Antibody and Antigen Construction, Expression, and Purification

The Kabat numbering scheme was used for identification of antibody variable and constant domain residues [24]. The anti-oxMIF antibody BaxM159 was isolated from a single-chain variable fragment (scFv) phage display library as described previously [22]. Heavy and light chain genes of BaxM159 and its variants were synthesized and cloned separately in mammalian expression vectors using standard cloning techniques. Antibodies were expressed in stably transfected Chinese hamster ovary (CHO) cell pools whose MIF gene had been knocked out by zinc-finger nuclease technology (Sigma Aldrich, Taufkirchen, Germany). Stable clone pools were generated by applying selective pressure (puromycin) for at least two weeks. Antibodies were purified from the cell culture supernatant by protein A chromatography as described previously [22,23]. Antibodies used in the pharmacokinetic study were polished by an additional cation exchange chromatography step to ensure that the injected material had a low aggregation level, i.e., <0.7% as evident by size-exclusion chromatography on HPLC system (SE-HPLC, see below). A Poros 50HS column (Thermo Fisher Scientific, Madison, CT, USA) was used for cation exchange chromatography using 30 mM sodium acetate pH 5.0 for binding and a gradient with 0–1 M NaCl, sodium acetate, pH 5.0, for elution. Antibodies were formulated eventually in glycine buffer, pH 5.0.

Recombinant MIF was expressed in *E. coli* and purified as described [23]. In brief, the MIF encoding gene was cloned into the pET16b expression vector (Novagen, Madison, WI, USA) and *E. coli* BL21 (Stratagene La Jolla, CA, USA) was transformed with the respective vector. Cells were lysed, and cell debris removed by centrifugation at 200,000× *g* for 20 min. Recombinant MIF was purified by application of the supernatent to DEAE-Sepharose FF (GE-Healthcare, Piscataway, NJ, USA) column followed by re-buffering in 20 mM Bis/Tris, pH 6.3 using a HiPrep 26/10 desalting column (GE-Healthcare). A second purification step employed cation exchange chromatography using a Source 30S (GE-Healthcare) column and elution of MIF with a linear salt gradient up to 100 mM NaCl.

### 2.2. Biochemical Analytics

MIF specific IgG titer was measured by enzyme-linked immunosorbent assay (ELISA). Typically, 96-well plates were coated with full length recombinant human MIF and incubated over night at +4 °C. Plates were then blocked by 1.5% fish gelatin diluted in phosphate-buffered saline (PBS), washed with PBS four times before applying samples in different dilutions. Samples were incubated 2 h at room temperature, the plates were washed four times with PBS, and human IgG was detected with a horseradish peroxidase-conjugated goat anti-human IgG (Sigma Aldrich, Taufkirchen, Germany). Finally, plates were washed four times with PBS before TMB (3,3′,5,5′-tetramethylbenzidine) was added. After incubation in the dark at room temperature for 30 min the reaction was stopped by addition of 1.8 M H_2_SO_4_. The amount of chromogenic product formed was determined spectrophotometrically at 450 nm.

Human IgG concentrations were determined by a specific ELISA. Typically, 1 µg/mL of an affinity purified anti-human-IgG Fc goat F(ab’)2 (Jackson Immuno Research Laboratories Inc., West Baltimore Pike, PA, USA) was coated onto microtiter plates, blocked with 2% bovine serum albumine diluted in Tris-buffered saline with Tween 20 (TBST) for 1 h and washed four times with phosphate-buffered saline with Tween 20 (PBST) before applying samples in different dilutions. After 1 h incubation at room temperature, the plates were washed four times with PBST. Total human IgG was detected by a horseradish peroxidase–conjugated goat F(ab’)2, specific against the human F(ab’)2 (Jackson Immuno Research Laboratories Inc., West Baltimore Pike, PA, USA). Subsequently, TMB was added and incubated at room temperature for 15 min. The reaction was stopped with 1.8 M H_2_SO_4_, and the reaction product was quantified as described above. Plasma concentration was calculated against standard curves of the test items. The human IgG ELISA was established as a fit for purpose assay.

OxMIF and total MIF was quantitatively determined by a differential ELISA as described [19]. Epitopes of the antibodies were mapped by monitoring binding to overlapping MIF-derived peptides as described [22].

The affinity of the antibodies was determined by surface plasmon resonance as described [25]. In brief, 40 RU units of each antibody was immobilized onto a CM5 sensor chip (GE-Healthcare). Recombinant MIF was converted to its oxidized form by treatment with 0.2% Proclin300 and injected into a BIAcore T200 device (GE-Healthcare) at concentrations of 3–25 nM in HBS-EP (HEPES 10 mM, NaCl 150 mM, EDTA 3mM, 0.005% Tween-20). After each cycle the chip was regenerated with 50 mM NaOH/1 M NaCl. Measurements were run at 25 °C and data were analyzed according to the 1:1 Langmuir model.

The thermal stability of the antibodies was evaluated by differential scanning fluorimetry and differential scanning calorimetry. Differential scanning fluorimetry was conducted by using the Protein Thermal Shift assay (Thermo Fisher) according the instructions of the supplier. Fluorescence measurements were done on the 7500 Fast RT-PCR System (Thermo Fisher). The protein Thermal Shift Software (Thermo Fisher) was used for data analysis. The data analysis was based on the invariant calculation method for determining the midpoint temperature of the first unfolding transition. Differential scanning calorimetry was conducted using a NanoDSC-III (Calorimetry Sciences Corporation, Waters Corporation, Milford, MA, USA) device with operation temperature range 25–100 °C and heating rate 2 °C/min in both the heating and cooling directions. After measuring both cells were filled with buffer and heated and cooled at the same settings as the sample run to obtain the buffer baseline. The buffer baseline was subtracted from the sample run and a polynomial baseline was generated to calculate the molar heat capacity.

Time dependent aggregation/fragmentation of the antibodies were analyzed by SE-HPLC (Agilent) using a TSKgel G3000SW column (Tosoh Biosciences, Tokyo, Japan). The column was eluted with 20 mM NaH_2_PO_4_, pH 6.8, 150 mM Na_2_SO_4_, 0.02% NaN3, and 10% dimethyl sulfoxide. The column effluent was monitored continuously at 280 nm. The data were evaluated with a chromatographic data system (System Karat, Beckman, Brea, CA, USA). The performance of the whole system was checked and monitored by a system suitability test using a molecular weight standard (Bio-Rad, Hercules, CA, USA).

### 2.3. Pharmacokinetics

Animal experiments were carried out in accordance with the guidelines of the Medical University of Vienna (Vienna, Austria; Good Scientific Practice Manual) and were approved by the Animal Welfare Committee of the Medical University of Vienna and the Austrian Science Ministry. The pharmacokinetic characteristics of the anti-MIF antibodies were determined in male MF1 nude mice (*n* = 4 for each condition). Antibodies were administered intravenously at doses of 5, 10, or 30 mg/kg. The selected doses covered the range at which these antibodies inhibited the growth of xenograft tumors in nude mice [21]. Blood was drawn at different time points into heparinized capillaries. Plasma was prepared by centrifugation and human IgG concentrations were determined by human IgG specific ELISA.

### 2.4. Statistics

Distributions were evaluated by the Kolmogorov-Smirnov test. If normal distribution was confirmed, data were evaluated by one-way ANOVA followed by Tukey’s post hoc test. Correlation analysis was based on Spearman’s correlation. Pharmacokinetic parameters were calculated by subjecting the data to a nonlinear least-squares fit to equations describing a mono- or a bi-exponential decay corresponding to a one- or two-compartment model, respectively. An F-test based on the extra sum of squares principle was used to confirm that the more complex model resulted in a statistically significant improvement (*p* < 0.05) in the fit. This was the case for all curves but those for 5 mg/kg parental BaxM159. Based on the bi-exponential decay model the following PK parameters were estimated: initial concentration, clearance (CL), area under the curve (AUC), terminal or beta phase half-life (t_1/2_,_β_), and volume of distribution at steady state (V_D,ss_). The AUC was estimated from the time-concentration curves based on the trapezoidal rule from 0 to 144 h. CL was calculated from the relation CL = D/AUC, where D and AUC are administered dose and area under the curve, respectively. The V_D,ss_ was calculated from the relation V_D,ss_ = CL × MRT, where CL and MRT are clearance and mean residence time.

## 3. Results

### 3.1. Design and Generation of Anti-oxMIF Antibody BaxM159 Variants

It is hypothesized that germline V genes have been optimized during evolution for high expression and stability. Hence, a given antibody sequence should match the germline V genes as close as possible. Based on this approach we identified the germline VL and VH genes with the highest homology to the BaxM159 VL and VH sequence, respectively, by data base alignments (NCBI IG (http://www.ncbi.nlm.nih.gov/igblast/)).

**Figure 1:**
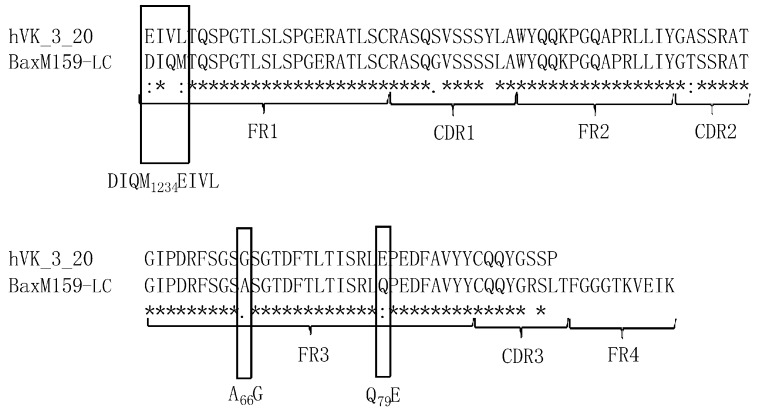
Comparison of BaxM159 VL sequence with germline VL subclass 3-20. Software clustal O was used for sequence alignment. * indicates identity, indicates similarity. Frame works, complementarity-determining region (CDR) identification, and amino acid numbering is indicated according to kabat scheme.

**Figure 2:**
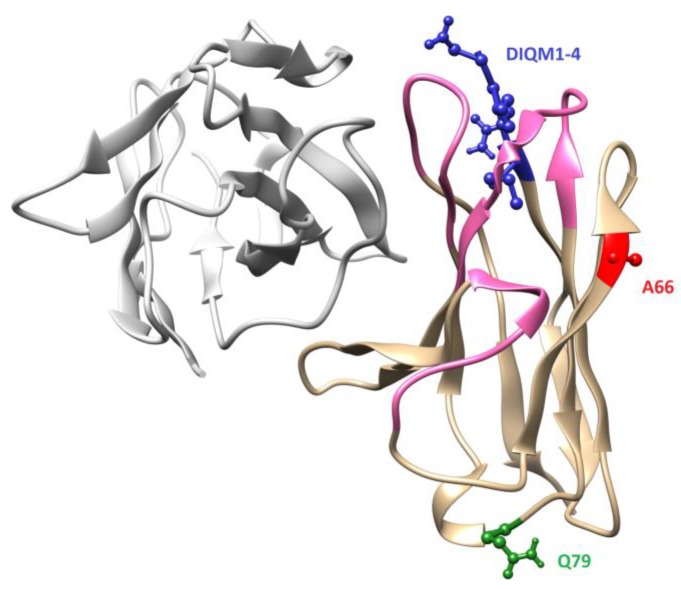
Three-dimensional structure model of the variable fragment (Fv) of BaxM159. The model was generated using the automated antibody modeling web-portal Kotai Antibody Builder (http://kotaiab.org/; [26]). Molecular graphics and analyses were performed with Vector NTI package. HC is gray, LC is beige, CDRs are pink colored. Amino acids DIQM1-4 are blue, A66 is red, Q79 is green colored.

We found that the BaxM159 VH framework was identical to germ line VH gene 3-23 framework (NCBI germline M99660) (Supplementary Figure S1). Accordingly, based on the evolutionary argument, any sequence adaption of BaxM159 VH framework is predicted to be futile. The germline VL(κ) gene 3-20 was identified to be the germline VL gene with the highest homology (92.86% identity in framework region, 91.3% in total V region) to BaxM159 VL in the framework region (Figure 1). The framework of BaxM159 VL differs from germline VL gene 3-20 at three locations, i.e. at amino acid position 1 to 4, 66, and 79. BaxM159 VL has the sequence DIQM at positions 1–4, an alanine (A) at position 66 and a glutamate (Q) at position 79. In contrast, germline VL gene 3-20 has the sequence EIVL at positions 1 to 4, a glycine (G) at position 66 and a glutamic acid (E) at position 79. Based on the amino acid sequence of BaxM159 we generated a three-dimensional structure model of the Fv (fragment variable) using the automated antibody modeling web-portal Kotai Antibody Builder (http://kotaiab.org/; [26]). The model revealed that all three regions DIQM1-4, A66, and Q79 are surface-exposed (Figure 2). This is in agreement with the high-to-medium side chain solvent accessibility of the amino acid residues at these positions according to an analysis of approx. 400 antibody structures [27]. Moreover, we analyzed the non-matching amino acids for their frequency at their particular position in the context of all known framework sequences to date by using the web-based antibody database abysis (http://www.abysis.org/; [28]). Indeed, an alanine at position 66 has a frequency of 0.5% in the interrogated dataset, making it an unusual residue. In contrast, glycine has a frequency of 61% at position 66.

Next, we generated BaxM159 variants with DIQM1-4EIVL, A66G, or Q79E mutations, respectively, or combinations thereof (Table 1). In total, nine different variants of BaxM159 were generated. Of note, the heavy chain sequence was unchanged in BaxM159 variants compared to the parental BaxM159 heavy chain sequence except that the C-terminal lysine was deleted from the sequence. The C-terminal lysine was omitted since it is clipped frequently during production of antibodies [29]. The clipping of the C-terminal lysine may increase antibody product heterogeneity which is to be avoided. To maintain comparability with previous data, two versions of parental antibody BaxM159 were generated: 2-DIQMAQ-K which harbors a C-terminal lysine and 1-DIQMAQ which does not contain the C-terminal lysine.

### 3.2. Framework Optimized BaxM159 Variants Have Unaltered Epitope Binding Properties

It is essential that the sequence-optimized BaxM159 variants recognize the target epitope with affinity and specificity similar to the parental BaxM159. Hence, we evaluated the BaxM159 variants for their binding to the target oxMIF by ELISA, their epitope specificity by monitoring binding to overlapping MIF-derived peptides and their affinity for oxMIF by surface plasma resonance. The equilibrium dissociation constant K_D_ of all BaxM159 variants did not differ by more than a factor of two from that of the parental antibody BaxM159 (Table 2). Similarly, the ka and kd of all variants varied between 1.3 × 10^5^–3.1 × 10^5^ and 2.9 × 10^−4^–6.4 × 10^−4^, respectively; i.e., ka and kd of all variants were comparable. Hence, we conclude that the affinity and binding kinetics of the BaxM159 variants was not altered to an appreciable extent by their mutation in the framework region. Similarly, BaxM159 variants bound oxidized MIF but not reduced MIF (data not shown) as the parental antibodies [19]. Therefore, the oxMIF specificity was not altered by the mutations in the framework region. Moreover, all BaxM159 variants recognized the MIF epitope ERLRISPDRVYINYYDM (MIF amino acid positions 82–108; data not shown), as described previously [22]. Thus, all BaxM159 variants had the same MIF epitope specificity as the parental antibodies.

### 3.3. Framework Optimization of Anti-oxMIF Antibody BaxM159 Increases Expression

**Figure 3:**
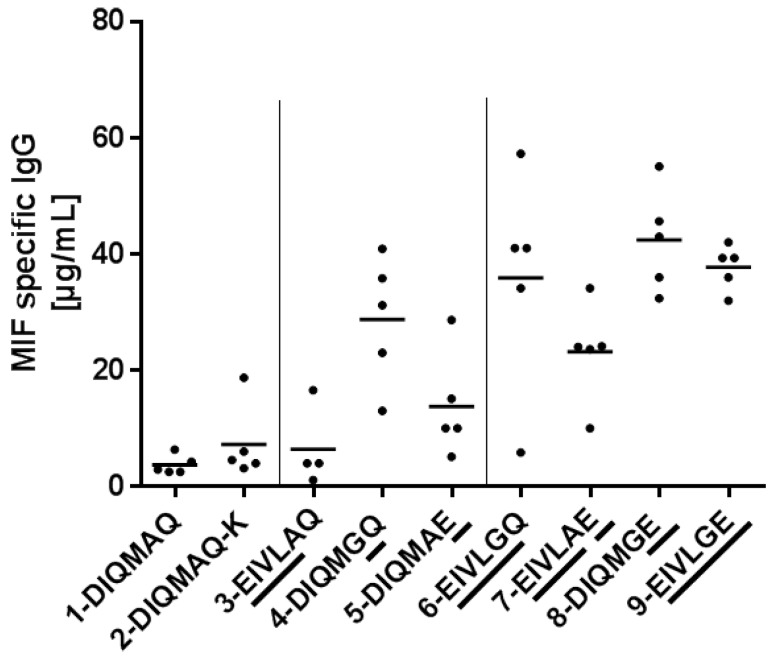
Macrophage migration inhibitory factor (MIF)-specific IgG titer of BaxM159 variants. MIF-specific IgG titer was determined by ELISA. BaxM159 variants were grown to ml scale starting with an initial cell number of 0.3–0.5 × 10^6^ cell/mL. At day 7–8 of cell cultivation MIF-specific IgG titer was determined from cell culture supernatant by ELISA. In total, five independent expression experiments were conducted. Statistical analysis is given in the Supplement Table S1.

In order to determine the expression of BaxM159 variants clone pools were grown at ml scale. After 7–8 days of cell cultivation, the MIF-specific IgG titer was determined by ELISA. In total, five independent expression experiments were conducted. As described earlier, three different BaxM159 variants were generated which had mutations in a single region, i.e., variant DIQM1-4EIVL (3-EIVLAQ), or at single position, i.e., A66G (4-DIQMGQ), and Q79E (5-DIQMAE). The variant 4-DIQMGQ with the A66G mutation was the only one of these three which increased the titer by factor 3–5 compared to the parental BaxM159 controls (Figure 3). In contrast, two variants 3-EIVLAQ or 5-DIQMAE did not increase the titer significantly compared to the parental BaxM159. Moreover, the variant 4-DIQMGQ had a significantly higher titer than the variants 3-EIVLAQ and 5-DIQMAE.

**Figure 4:**
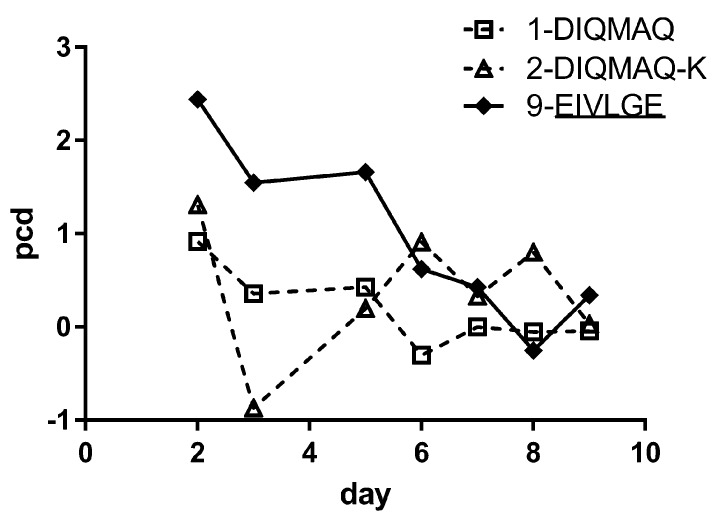
Specific productivity of BaxM159 variants at 10 L bioreactor scale. Specific productivity pcd is calculated as pcd = (pg × cell^−1^ × day^−1^). IgG titer of the 10 L bioreactor scale experiment is shown in the Supplementary Figure S2.

Only BaxM159 variants which included the A66G mutation in combination with other mutations (9-EIVLGE, 6-EIVLGQ, 8-DIQMGE) increased the titer significantly by factor 4–10 compared to parental BaxM159. In contrast, the combination of mutants DIQM1-4EIVL and Q79E (7-EIVLAE) did not increased the titer significantly compared to parental BaxM159. Hence, the A66G mutation in the framework of BaxM159 seems to be crucial for increased expression of the antibody. The increase in expression was apparently caused by an increase in specific productivity (pg × cell/day) during the first five days of cell culture as evident from a 10 L batch run of 9-EIVLGE versus parental BaxM159 controls (Figure 4).

### 3.4. Framework Optimization of Anti-oxMIF Antibody BaxM159 Increases Stability and Decreases Aggregation Propensity

**Figure 5:**
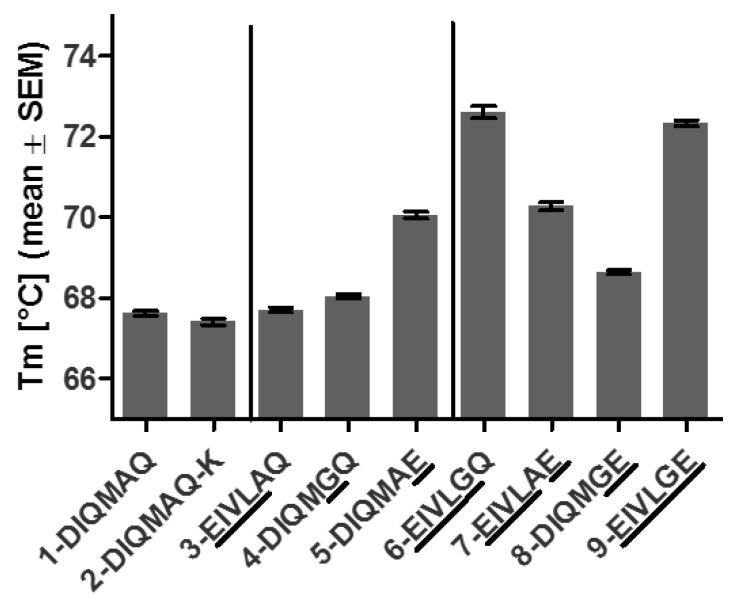
Thermal stability of BaxM159 variants determined by differential scanning fluorimetry. Standard error mean (SEM) is indicated by whiskers**.**

To assess the thermal stability of BaxM159 variants, antibodies were evaluated by differential scanning fluorimetry (Figure 5) and differential scanning calorimetry (Supplementary Figures S3 and S4). Mutations in the single region DIQM1-4EIVL (3-EIVLAQ) or A66G (4-DIQMGQ) increased the protein melting temperature Tm slightly compared to parental BaxM159 antibodies. However, mutation Q79E (5-DIQMAE) had by far the most Tm enhancing effect of the single region mutations and increased the Tm by >2 °C over the Tm of parental BaxM159.

Combinations of Q79E with DIQM1-4EIVL or A66G (7-EIVLAE or 8-DIQMGE) did not increase Tm further compared to single mutation Q79E alone (5-DIQMAE). In contrast, combining DIQM1-4EIVL and A66G (6-EIVLGQ) or DIQM1-4EIVL, A66G and Q79E (9-EIVLGE) had a synergistic effect, i.e., increased Tm compared to the variants with mutations in a single region (3-EIVLAQ, 4-DIQMGQ, 5-DIQMAE). Hence, 6-EIVLGQ and 9-EIVLGE had the highest Tm of all BaxM159 variants with an increase of >4 °C compared to the parental BaxM159. All findings, which were obtained by differential scanning fluorimetry, were confirmed by differential scanning calorimetry (Supplementary Figures S3 and S4).

**Figure 6:**
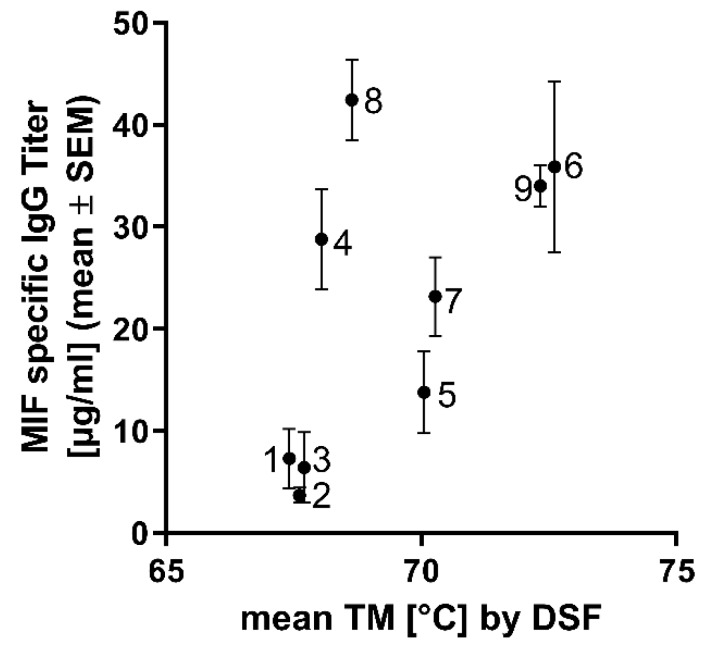
Correlation between thermal stability (Tm) of BaxM159 variants and MIF specific IgG titer. Thermal stability was determined by differential scanning fluorimetry (DSF). Standard error mean (SEM) of MIF specific titer is indicated by whiskers. Numbers indicate the ID of the antibody variants as described in Table 1.

It is reasonable to assume that the yield of functional protein is also determined by its stability. Accordingly, we examined if there was a correlation between MIF-specific IgG titer and the mean Tm of each BaxM159 variant: it is evident from the plot shown in Figure 6 that there is a significant correlation between the protein stability Tm and IgG titer (*p* = 0.043 based on Spearman’s rank-order correlation). This was also seen when the Tm-estimates obtained by differential scanning calorimetry were plotted against MIF-specific IgG titer (data not shown).

**Figure 7:**
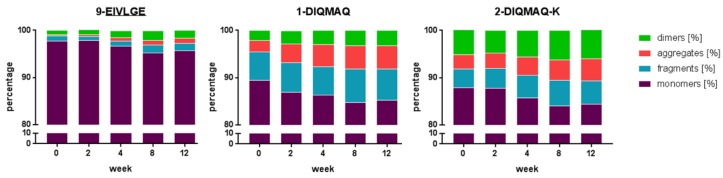
Aggregation/fragmentation of BaxM159 variants determined by size-exclusion chromatography (SEC) over a time course of 12 weeks at 4 °C.

Aggregation and fragmentation are undesirable phenomena in antibody production and formulation. Hence, we selected the variant 9-EIVLGE for further characterization because of its desirable properties, i.e., it could be produced at high titers and it displayed high thermal stability. We monitored the propensity of variant 9-EIVLGE to form aggregates over a time period of 12 weeks at 4 °C in comparison to parental BaxM159: samples were analyzed by SE-HPLC after 0, 2, 4, 8, and 12 weeks (Figure 7). At the start, the monomer content of variant 9-EIVLGE exceeded 97%. Furthermore, the monomer content did not drop below 95% within the twelve weeks of the observation period. In contrast, parental BaxM159 had a monomer content of <90% on day 0 and showed a further decrease down to 85%. This decrease in monomer content was accompanied by an increase in dimers and high molecular weight aggregates. In addition, we also observed a sizable fraction of fragmented BaxM159, which only increased modestly over the 12-week observation period. Hence, the framework optimized 9-EIVLGE showed a lower propensity of aggregation during storage compared to the parental BaxM159 antibodies. Even after 12 weeks storage at 4 °C 9-EIVLGE had a higher percentage of monomeric IgG than parental BaxM159 antibodies at the start of the study.

### 3.5. Framework Optimization of BaxM159 Does not Alter the In Vitro Functionality but Improves Its Pharmacokinetics

Based on its high MIF-specific IgG titer and high thermal stability the variant 9-EIVLGE was used for in-depth functional analysis in vitro and in vivo. The functional potency of anti-MIF antibodies was tested by a chemokinesis assay. The chemokinesis assay revealed an IC_50_ of 0.7 nM for variant 9-EIVLGE, which matched the potency of parental BaxM159 of 0.7 nM ± 0.2. Therefore, framework optimization of BaxM159 did not affect the in vitro potency.

**Figure 8:**
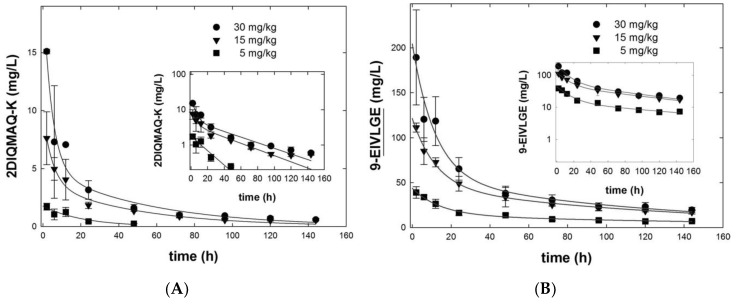
Pharmacokinetics of parental BaxM159 2-DIQMAQ-K (**A**) and BaxM159 variant 9-EIVLGE (**B**) after intravenous administration to MF1 nude mice. BaxM159 variants were injected intravenously via the tail vein into male MF1 nude mice (*n* = 4) at doses of 5, 10, 30 mg/kg. Blood (80 µL) was drawn at the indicated time points and centrifuged to generate plasma. The concentration of human IgG was determined by ELISA in these plasma samples. The solid lines in panel (**A**,**B**) were drawn by fitting the data points to the equation for a biexponential decay/two-compartment model with the notable exception of the curve for 5 mg/kg parental BaxM159 2-DIQMAQ-K (squares in (**A**)). It is evident that the concentrations of the 5 mg/kg parental BaxM159 2-DIQMAQ-K were below the detection limit at time points > 48 h. Hence, there were not enough data points and the data were only fitted to the equation for a monoexponential decay. The insets show the logarithmic transformation of the data to illustrate the presence of two components.

We compared the pharmacokinetics of anti-MIF antibodies 9-EIVLGE and parental BaxM159 (2-DIQMAQ-K) after intravenous injection of three different doses into MF-1 nude mice. Antibodies used in the pharmacokinetic study were polished by an additional cation exchange chromatography step resulting in removal of aggregates below 0.7% as confirmed by SEC-HPLC. Plasma samples were taken over a period of up to 144 h and analyzed for human IgG levels by ELISA. At equivalent doses, variant 9-EIVLGE reached about 10-fold higher initial concentrations in the blood than parental BaxM159 (2-DIQMAQ-K); this difference was maintained throughout the observation (panels A and B in Figure 8). In addition, the concentration of variant 9-EIVLGE decreased more slowly than parental 2-DIQMAQ-K as evident by the terminal half-life t½, β (Table 3) of about 4.5 days (dose-averaged). In fact, the terminal half-life t½, β of 9-EIVLGE was typical of IgG (four to six days). In contrast, the terminal half-life t½, β of parental 2-DIQMAQ-K was only about 1.5 days. Accordingly, the clearance of parental 2-DIQMAQ-K was more than an order of magnitude larger than that of variant 9-EIVLGE. Conversely, the exposure (AUC) of variant 9-EIVLGE was about 20-fold higher than of parental 2-DIQMAQ-K (Table 3). Moreover, variant 9-EIVLGE had a 20-fold lower volume of distribution at steady state (V_D,ss_) compared to parental 2-DIQMAQ-K. Similar differences were seen if the antibodies were administered by subcutaneous injection (data not sown). Furthermore, variant 9-EIVLGE exposure AUC was dose depended in the tested dose range whereas CL and V_D,ss_ were dose-independent. In summary, variant 9-EIVLGE has superior pharmacokinetic characteristics compared to parental BaxM159 (2-DIQMAQ-K).

## 4. Discussion

Efficacy, safety, and developability of therapeutic antibodies depend—at least in part—on their biopharmaceutical properties such as stability, aggregation propensity, and expression levels. Here, we improved the biopharmaceutical properties of the anti-oxMIF antibody BaxM159 by introducing framework mutations.

All BaxM159 variants with the individual mutations DIQM1-4EIVL, A66G, Q79E, or combinations thereof had the same oxMIF specificity, oxMIF affinity, and in vitro potency in a cell-based assay as the parental BaxM159. This is in line with a previous analysis of approximately 400 antibody structures [27]. This study showed that the framework positions that have been mutated here contribute only modestly or not at all to the VL/antigen interface. Hence, mutations at those framework positions should not affect the antigen-antibody binding and therefore should not modulate affinity or specificity. However, indirect effects have been appreciated: residues outside of the CDRs can contribute indirectly to antigen binding, e.g., amino acids within the Vernier zone may have a critical role in supporting the loop structures of the CDRs [30]. In fact, M4/L4 and A66/G66 are part of the Vernier zone. Nevertheless, the selected positions tolerated the substitutions without any negative impact on specificity, affinity, or in vitro potency, although two of the positions are part of the Vernier zone.

The A66G mutation was the crucial determinant for an increase in titer, regardless of whether it was introduced as a single mutation or in combination with mutations in other regions. However, the mutation A66G improved stability only to a modest extent. A sequence analysis revealed that alanine at position 66 is rare (0.5%), whereas glycine is very common in both germline VL genes and rearranged antibody sequences. This might be due to a positive phi torsion angle at position 66 within this loop, which is feasible for glycine [12]. The structural variability at the position 66 is low with ≤0.5Å (average rms deviation from mean Cα position) according to an analysis of about 400 antibody structures [27]. Moreover, position 66 packs together with other residues to form the upper core of the V domain and can influence the orientation and the conformation of the CDR-L1 loop by interacting with side chains of position 27d [12,27,31,32]. Hence, position 66 is predicted to be of structural importance but thermal stability is affected to a surprisingly little by the mutation A66G evaluated here.

Of all single region mutations (3-EIVLAQ, 4-DIQMGQ, 5-DIQMAE), the Q79E mutation improved thermal stability most. The main-chain carbonyl group of E79 has been described to form a hydrogen bond with R61 of the LC chain [31]. The increased stability may be caused by re-establishing this hydrogen bond in the Q79E mutant. However, we did not observe any impact on expression by the Q79E mutation.

Combining the single region mutations (3-EIVLAQ, 4-DIQMGQ, 5-DIQMAE) resulted in further increase of thermal stability and expression. Indeed, the combination of all three mutation regions (9-EIVLGE) achieved the highest antibody titer and nearly the best stability. Similarly, combining selected framework (or CDR) mutations within the VH or VL domains has been described to improve biopharmaceutical properties additively or even synergistically [9,12,14,17].

Correlating the expression titer with the thermal stability of all generated mutations or mutation combinations revealed a low but significant association. Moreover, BaxM159 variant 9-EIVLGE that, combined all three mutation regions, had reduced aggregation propensity compared to the parental BaxM159. Other groups have also found a simultaneous increase in expression and stability in general [33] or upon rational mutation in the VH or VL domains [11,12,34]. Similarly, an inverse association between expression and aggregate formation was observed when comparing V domain mutations [9,11]. Aggregate formation has been also reported to correlate negatively with thermal stability [10]. In contrast, no strict association between thermal stability and aggregate formation [35,36] or thermal stability and shelf-life [34] was found in other V domain mutational studies. The conflicting reports regarding correlation of aggregate formation, expression and stability is reflected in our data. On the one hand, an overall correlation of stability and expression was observed. On the other hand, the single region mutation A66G enhanced expression but only faintly stability. Vice versa, Q79E improved stability but only weakly titer. Hence, a reliable prediction of expression by stability measurement seems difficult, at least for V domain engineering. Possibly, aggregation decreasing mutations modify aggregation-prone unfolded or partially folded states and do not improve thermal stability as suggested elsewhere [37,38]. More studies are required to understand and predict the effect of V domain engineering on biopharmaceutical properties.

Based on the superior expression, stability, and aggregation propensity we focused on the BaxM159 variant 9-EIVLGE which combined all three mutation regions for studying the pharmacokinetics. When compared to the parental control, dose-normalized exposure over time (AUC) and the circulating levels of 9-EIVLGE was ≥10-fold increased. Possibly the higher propensity to aggregate and the lower stability of parental BaxM159 resulted in the enhanced formation of immune complexes, which are cleared rapidly. This interpretation is supported by the very large clearance and volume of distribution at steady state estimates for BaxM159. In contrast, clearance of 9-EIVLGE was ≥10-fold lower and the terminal half-live of 9-EIVLGE increased compared to parental BaxM159. In fact, 9-EIVLGE was cleared with elimination kinetics typical of IgG. It is likely that the improved pharmacokinetics of variant 9-EIVLGE are not due to different disposition of the two antibodies since healthy mice as used in this PK study do not express oxMIF and no target dependent interaction with murine leukocytes can be expected [19]. Pharmacokinetics of therapeutic antibodies have been improved previously by PEGylation, zwitterionic gel encapsulation [39], Fc engineering, polysialylation, amino acid polymers, and albumin-binding derivatives [40], but also by CDR engineering [41]. Here we were able to improve the pharmacokinetics by V domain framework engineering in association with enhanced biopharmaceutical properties. Indeed, the improved pharmacokinetic behavior of variant 9-EIVLGE might result in improved efficacy in vivo compared to the parental BaxM159 even if the in vitro potency of both molecules is similar.

Framework modifications have been shown to improve biophysical and biopharmaceutical properties of antibodies. Framework modifications can be based on knowledge-based rules, structural analysis, or statistical analysis of antibody sequences [8,13,14,17,34,42,43,44,45]. Buchanan et al. [11] and Chen et al. [46] described germline based framework modification of antibodies but did not report enhanced biopharmaceutical properties. In contrast, identifying and combining germline derived frame work variations in the V domain of BaxM159 resulted in >5-fold increased IgG concentration, an increased thermal stability, and a reduced tendency to aggregate. Since the latter two parameters are mainly molecule related, a similar improvement might be expected if any other mammalian cell systems than CHO cells would be used for expression. Importantly, the identified mutations did not alter the functionality of BaxM159 in vitro but improved the pharmacokinetic profile in vivo. Dobson et al. [41] improved the pharmacokinetic behavior of a human antibody, but this required CDR modifications based on experimental structure analysis, i.e., hydrogen/deuterium exchange and cross-linking-mass spectrometry. The approach of germline-based framework modification does not require extensive experimental, structural or statistical sequence analysis. Hence, this approach might be suitable for optimization of antibodies in general.

## 5. Conclusions

Germline-based framework modifications were used to optimize the anti-oxMIF antibody BaxM159. The combined modifications at positions 1**–**4, 66, and 79 of the VL domain did not amend oxMIF affinity, oxMIF specificity or function of the antibody. However, the modifications improved thermal stability, aggregation propensity, and expression. Moreover, the framework modifications resulted in reduced clearance, prolonged half-life and higher exposure. Germline-based framework modifications have therefore the potential to enhance both biopharmaceutical properties and pharmacokinetics of antibodies in a simple but efficient approach. 

## Figures and Tables

**Table 1:** BaxM159 variants. Underlined amino acids in the BaxM159 variant name indicate amended amino acids in positions 1–4, 66, and 79. C-terminal lysine is removed unless K indicates presence of C-terminal lysine. Antibodies 1-DIQMAQ and 2-DIQMAQ-K represent the parental antibody. Amino acid numbering is indicated according to kabat scheme.

ID	ID and Name of BaxM159 Variant	Amino Acid at Positions 1–4	Amino Acid at Position 66	Amino Acid at Position 79
1	1-DIQMAQ	DIQM	A	Q
2	2-DIQMAQ-K	DIQM	A	Q
3	3-EIVLAQ	EIVL	A	Q
4	4-DIQMGQ	DIQM	G	Q
5	5-DIQMAE	DIQM	A	E
6	6-EIVLGQ	EIVL	G	Q
7	7-EIVLAE	EIVL	A	E
8	8-DIQMGE	DIQM	G	E
9	9-EIVLGE	EIVL	G	E

**Table 2:** Affinity of BaxM159 variants determined by surface plasma resonance. The surface plasma resonance analysis was repeated twice except for indicated antibody variants. Standard deviation of ka, kd, and K_D_ is indicated.

ID	ID and Name of BaxM159 Variant	ka (M^−1^s^−1^)	kd (s^−1^)	K_D_ (M)
1	1-DIQMAQ	1.1 × 10^5^	2.9 × 10^−4^	2.6 × 10^−9^
2	2-DIQMAQ-K	1.3 × 10^5^	3.8 × 10^−4^	3.0 × 10^−9^
3	3-EIVLAQ	2.0 × 10^5^	3.1 × 10^−4^	1.5 × 10^−9^
4	4-DIQMGQ	1.6 × 10^5^ ± 8.7 × 10^4^	5.6 × 10^−4^ ± 5.0 × 10^−4^	4.3 × 10^−9^ ± 2.6 × 10^−9^
5	5-DIQMAE	1.6 × 10^5^	4.0 × 10^−4^	2.5 × 10^−9^
6	6-EIVLGQ	2.6 × 10^5^ ± 1.2 × 10^5^	6.4 × 10^−4^ ± 3.5 × 10^−5^	2.7 × 10^−9^ ± 1.1 × 10^−9^
7	7-EIVLAE	1.8 × 10^5^ ± 6.3 × 10^3^	4.4 × 10^−4^ ± 1.5 × 10^−4^	2.4 × 10^−9^ ± 7.6 × 10^−10^
8	8-DIQMGE	1.3 × 10^5^	5.4 × 10^−4^	4.2 × 10^−9^
9	9-EIVLGE	3.1 × 10^5^ ± 6.5 × 10^4^	4.4 × 10^−4^ ± 2.2 × 10^−4^	1.4 × 10^−9^ ± 4.1 × 10^−10^

**Table 3:** Pharmacokinetic parameters of antibody 9-EIVLGE and parental antibody 2-DIQMAQ-K derived from an in vivo study in male MF1 nude mice. The kinetic parameters initial concentration (C_0_), clearance CL, exposure (AUC_0–144_ = area under the curve from 0 to 144 h), terminal or beta phase half-life (t½, _β_), and volume of distribution at steady state (V_D,ss_), were calculated by fitting the data summarized in Figure 8 to an equation for biexponential decay (= two-compartment model), where the α-phase and the β-phase are assumed to reflect the distribution phase (i.e., the transfer from a central to a peripheral compartment) and the elimination, respectively. Note that for the dose of 5 mg/kg of parental 2-DIQMAQ-K the concentrations at time intervals > 48 h were below the detection limit; hence, kinetic estimates could not be determined (n.d.). CL and V_D,ss_ were adjusted for mouse body weight (30 g). Data represent parameter estimates and their standard errors.

Antibody	2-DIQMAQ-K			9-EIVLGE		
Dose (mg/kg)	5	15	30	5	15	30
C_0,α_ (mg/L)	n.d.	6.0 ± 1.1	15.6 ± 3.1	29.7 ± 3.42	76.4 ± 7.3	149.1 ± 33.4
C_0,β_ (mg/L)	n.d.	3.7 ± 1.1	5.0 ± 2.5	14.0 ± 3.68	45.1 ± 7.9	55.2 ± 35.9
t½, _β_ (h)	n.d.	34.5	37.9	135.9	96.3	87.7
AUC_(0–144h)_ iv (mg/L·h)	n.d.	209.6	326.8	1873.6	5036.7	6611.3
Clearance CL (mL/h/kg)	n.d.	72 ± 13	98 ± 16	3.5 ± 1.4	3.2 ± 1.4	3.6 ± 0.8
V_D,ss_ (mL/kg)	n.d.	3067 ± 710	3757 ± 957	170 ± 40	160 ± 23	190 ± 33

## Supplementary Materials

The following are available online at https://www.mdpi.com/2073-4468/8/3/46/s1. Table S1: Statistical analysis of MIF specific IgG titer of BaxM159 variants, Figure S1: Comparison of BaxM159 VH sequence with germline VH subclass 3–23. Software clustal O was used for sequence alignment. * indicates identity, indicates similarity. Frame works, CDR identification and amino acid numbering is indicated according to kabat scheme, Figure S2: IgG titer of indicated BaxM159 variants at 10 L bioreactor scale. IgG titer was determined by ELISA, Figure S3: Thermal stability of BaxM159 variants determined by differential scanning calorimetry. Standard error mean (SEM) is indicated by whiskers, Figure S4: Differential scanning calorimetry thermograms of BaxM159 variants 1-DIQMAQ, 2-DIQMAQ-K and 9-EIVLGE.

## References

[1] Ecker D.M., Jones S.D., Levine H.L. (2015). The therapeutic monoclonal antibody market. mAbs.

[2] Rita Costa A., Elisa Rodrigues M., Henriques M., Azeredo J., Oliveira R. (2010). Guidelines to cell engineering for monoclonal antibody production. Eur. J. Pharm. Biopharm..

[3] Lu Z.J., Deng S.J., Huang D.G., He Y., Lei M., Zhou L., Jin P. (2012). Frontier of therapeutic antibody discovery: The challenges and how to face them. World J. Biol. Chem..

[4] Buttel I.C., Chamberlain P., Chowers Y., Ehmann F., Greinacher A., Jefferis R., Kramer D., Kropshofer H., Lloyd P., Lubiniecki A. (2011). Taking immunogenicity assessment of therapeutic proteins to the next level. Biologicals.

[5] Moussa E.M., Panchal J.P., Moorthy B.S., Blum J.S., Joubert M.K., Narhi L.O., Topp E.M. (2016). Immunogenicity of Therapeutic Protein Aggregates. J. Pharm. Sci..

[6] Perchiacca J.M., Tessier P.M. (2012). Engineering aggregation-resistant antibodies. Annu. Rev. Chem. Biomol. Eng..

[7] Ratanji K.D., Derrick J.P., Dearman R.J., Kimber I. (2014). Immunogenicity of therapeutic proteins: Influence of aggregation. J. Immunotoxicol..

[8] Igawa T., Tsunoda H., Kuramochi T., Sampei Z., Ishii S., Hattori K. (2011). Engineering the variable region of therapeutic IgG antibodies. mAbs.

[9] Van der Kant R., Karow-Zwick A.R., Van Durme J., Blech M., Gallardo R., Seeliger D., Assfalg K., Baatsen P., Compernolle G., Gils A. (2017). Prediction and Reduction of the Aggregation of Monoclonal Antibodies. J. Mol. Biol..

[10] Fukuda J., Iwura T., Yanagihara S., Kano K. (2015). Factors to Govern Soluble and Insoluble Aggregate-formation in Monoclonal Antibodies. Anal. Sci..

[11] Buchanan A., Clementel V., Woods R., Harn N., Bowen M.A., Mo W., Popovic B., Bishop S.M., Dall’Acqua W., Minter R. (2013). Engineering a therapeutic IgG molecule to address cysteinylation, aggregation and enhance thermal stability and expression. mAbs.

[12] Ewert S., Honegger A., Pluckthun A. (2003). Structure-based improvement of the biophysical properties of immunoglobulin VH domains with a generalizable approach. Biochemistry.

[13] Tiller K.E., Tessier P.M. (2015). Advances in Antibody Design. Annu. Rev. Biomed. Eng..

[14] Monsellier E., Bedouelle H. (2006). Improving the stability of an antibody variable fragment by a combination of knowledge-based approaches: Validation and mechanisms. J. Mol. Biol..

[15] Potapov V., Cohen M., Schreiber G. (2009). Assessing computational methods for predicting protein stability upon mutation: Good on average but not in the details. Protein Eng. Des. Sel..

[16] Mason M., Sweeney B., Cain K., Stephens P., Sharfstein S.T. (2012). Identifying bottlenecks in transient and stable production of recombinant monoclonal-antibody sequence variants in Chinese hamster ovary cells. Biotechnol. Prog..

[17] Miller B.R., Demarest S.J., Lugovskoy A., Huang F., Wu X., Snyder W.B., Croner L.J., Wang N., Amatucci A., Michaelson J.S. (2010). Stability engineering of scFvs for the development of bispecific and multivalent antibodies. Protein Eng. Des. Sel..

[18] Steipe B., Schiller B., Pluckthun A., Steinbacher S. (1994). Sequence statistics reliably predict stabilizing mutations in a protein domain. J. Mol. Biol..

[19] Thiele M., Kerschbaumer R.J., Tam F.W., Volkel D., Douillard P., Schinagl A., Kuhnel H., Smith J., McDaid J.P., Bhangal G. (2015). Selective Targeting of a Disease-Related Conformational Isoform of Macrophage Migration Inhibitory Factor Ameliorates Inflammatory Conditions. J. Immunol..

[20] Schinagl A., Kerschbaumer R.J., Sabarth N., Douillard P., Scholz P., Voelkel D., Hollerweger J.C., Goettig P., Brandstetter H., Scheiflinger F. (2018). Role of the Cysteine 81 Residue of Macrophage Migration Inhibitory Factor as a Molecular Redox Switch. Biochemistry.

[21] Schinagl A., Thiele M., Douillard P., Volkel D., Kenner L., Kazemi Z., Freissmuth M., Scheiflinger F., Kerschbaumer R.J. (2016). Oxidized macrophage migration inhibitory factor is a potential new tissue marker and drug target in cancer. Oncotarget.

[22] Kerschbaumer R.J., Rieger M., Volkel D., Le Roy D., Roger T., Garbaraviciene J., Boehncke W.H., Mullberg J., Hoet R.M., Wood C.R. (2012). Neutralization of macrophage migration inhibitory factor (MIF) by fully human antibodies correlates with their specificity for the beta-sheet structure of MIF. J. Biol. Chem..

[23] Hussain F., Freissmuth M., Volkel D., Thiele M., Douillard P., Antoine G., Thurner P., Ehrlich H., Schwarz H.P., Scheiflinger F. (2013). Human anti-macrophage migration inhibitory factor antibodies inhibit growth of human prostate cancer cells in vitro and in vivo. Mol. Cancer Ther..

[24] Kabat E.A., Wu T.T. (1991). Identical V region amino acid sequences and segments of sequences in antibodies of different specificities. Relative contributions of VH and VL genes, minigenes, and complementarity-determining regions to binding of antibody-combining sites. J. Immunol..

[25] Hollriegl W., Bauer A., Baumgartner B., Dietrich B., Douillard P., Kerschbaumer R.J., Hobarth G., McKee J.S., Schinagl A., Tam F.W.K. (2018). Pharmacokinetics, disease-modifying activity, and safety of an experimental therapeutic targeting an immunological isoform of macrophage migration inhibitory factor, in rat glomerulonephritis. Eur. J. Pharmacol..

[26] Yamashita K., Ikeda K., Amada K., Liang S., Tsuchiya Y., Nakamura H., Shirai H., Standley D.M. (2014). Kotai Antibody Builder: Automated high-resolution structural modeling of antibodies. Bioinformatics.

[27] Honegger A., Pluckthun A. (2001). Yet another numbering scheme for immunoglobulin variable domains: An automatic modeling and analysis tool. J. Mol. Biol..

[28] Swindells M.B., Porter C.T., Couch M., Hurst J., Abhinandan K.R., Nielsen J.H., Macindoe G., Hetherington J., Martin A.C. (2017). abYsis: Integrated Antibody Sequence and Structure-Management, Analysis, and Prediction. J. Mol. Biol..

[29] Harris R.J. (1995). Processing of C-terminal lysine and arginine residues of proteins isolated from mammalian cell culture. J. Chromatogr. A.

[30] Foote J., Winter G. (1992). Antibody framework residues affecting the conformation of the hypervariable loops. J. Mol. Biol..

[31] Ewert S., Huber T., Honegger A., Pluckthun A. (2003). Biophysical properties of human antibody variable domains. J. Mol. Biol..

[32] Honegger A. (2008). Engineering antibodies for stability and efficient folding. Handb. Exp. Pharmacol..

[33] Jain T., Sun T., Durand S., Hall A., Houston N.R., Nett J.H., Sharkey B., Bobrowicz B., Caffry I., Yu Y. (2017). Biophysical properties of the clinical-stage antibody landscape. Proc. Natl. Acad. Sci. USA.

[34] Seeliger D., Schulz P., Litzenburger T., Spitz J., Hoerer S., Blech M., Enenkel B., Studts J.M., Garidel P., Karow A.R. (2015). Boosting antibody developability through rational sequence optimization. mAbs.

[35] Dudgeon K., Rouet R., Kokmeijer I., Schofield P., Stolp J., Langley D., Stock D., Christ D. (2012). General strategy for the generation of human antibody variable domains with increased aggregation resistance. Proc. Natl. Acad. Sci. USA.

[36] Perchiacca J.M., Bhattacharya M., Tessier P.M. (2011). Mutational analysis of domain antibodies reveals aggregation hotspots within and near the complementarity determining regions. Proteins.

[37] Jespers L., Schon O., Famm K., Winter G. (2004). Aggregation-resistant domain antibodies selected on phage by heat denaturation. Nat. Biotechnol..

[38] Chiti F., Stefani M., Taddei N., Ramponi G., Dobson C.M. (2003). Rationalization of the effects of mutations on peptide and protein aggregation rates. Nature.

[39] Zhang P., Sun F., Tsao C., Liu S., Jain P., Sinclair A., Hung H.C., Bai T., Wu K., Jiang S. (2015). Zwitterionic gel encapsulation promotes protein stability, enhances pharmacokinetics, and reduces immunogenicity. Proc. Natl. Acad. Sci. USA.

[40] Constantinou A., Chen C., Deonarain M.P. (2010). Modulating the pharmacokinetics of therapeutic antibodies. Biotechnol. Lett..

[41] Dobson C.L., Devine P.W., Phillips J.J., Higazi D.R., Lloyd C., Popovic B., Arnold J., Buchanan A., Lewis A., Goodman J. (2016). Engineering the surface properties of a human monoclonal antibody prevents self-association and rapid clearance in vivo. Sci. Rep..

[42] Haidar J.N., Yuan Q.A., Zeng L., Snavely M., Luna X., Zhang H., Zhu W., Ludwig D.L., Zhu Z. (2012). A universal combinatorial design of antibody framework to graft distinct CDR sequences: A bioinformatics approach. Proteins.

[43] Schaefer J.V., Pluckthun A. (2012). Engineering aggregation resistance in IgG by two independent mechanisms: Lessons from comparison of Pichia pastoris and mammalian cell expression. J. Mol. Biol..

[44] Jordan J.L., Arndt J.W., Hanf K., Li G., Hall J., Demarest S., Huang F., Wu X., Miller B., Glaser S. (2009). Structural understanding of stabilization patterns in engineered bispecific Ig-like antibody molecules. Proteins.

[45] Honegger A., Malebranche A.D., Rothlisberger D., Pluckthun A. (2009). The influence of the framework core residues on the biophysical properties of immunoglobulin heavy chain variable domains. Protein Eng. Des. Sel..

[46] Chen W., Li W., Ying T., Wang Y., Feng Y., Dimitrov D.S. (2015). Germlining of the HIV-1 broadly neutralizing antibody domain m36. Antiviral Res..

