# Supplementary material for: Optimization of an Antibody Light Chain Framework Enhances Expression, Biophysical Properties and Pharmacokinetics
Source: Antibodies (Basel). 2019 Sep 6;8(3):46. doi: 10.3390/antib8030046 (PMC6784111; doi:10.3390/antib8030046)
Supplement: Supplementary file 1 [file antibodies-08-00046-s001.pdf]

Supplement

Table S1. Statistical analysis of MIF specific IgG titer of BaxM159 variants. In total, five independently repeated experiments were conducted. Statistical significance was calculated by 1-way Anova test followed by Tukey. *p<0.05; **p<0.01; ***p<0.001; ****p<0.0001, ns: non significant.

	**1-DIQMAQ**	**2-DIQMAQ-K**	**3-EIVLAQ**	**4-DIQMGQ**	**5-DIQMAE**	**6-EIVLGQ**	**7-EIVLAE**	**8-DIQMGE**	**9-EIVLGE**
1-DIQMAQ		ns	ns	**	ns	***	ns	****	****
2-DIQMAQ-K	ns		ns	*	ns	**	ns	****	***
3-EIVLAQ	ns	ns		*	ns	**	ns	****	***
4-DIQMGQ	**	*	*		ns	ns	ns	ns	ns
5-DIQMAE	ns	ns	ns	ns		*	ns	**	**
6-EIVLGQ	***	**	**	ns	*		ns	ns	ns
7-EIVLAE	ns	ns	ns	ns	ns	ns		ns	ns
8-DIQMGE	****	****	****	ns	**	ns	ns		ns
9-EIVLGE	****	***	***	ns	**	ns	ns	ns	

```
hVH3 23        EVQLLESGGGLVQPGGSLRLSCAASGFTFSSYAMSWVRQAPGKGLEWVSAISGSGGSTYYADSVKG
BaxM-159-HC    EVQLLESGGGLVQPGGSLRLSCAASGFTFSWYAMDWVRQAPGKGLEWVSGIYPSGGRTKYADSVKG
               ****************************** ***.**************.*  *** * *******
                         FR1               CDR1    FR2           CDR2

hVH3 23        RFTISRDNSKNTLYLQMNSLRAEDTAVYYCA-------------------
BaxM-159-HC    RFTISRDNSKNTLYLQMNSLRAEDTAVYYCARVNVIAVAGTGYYYYGMDV
               *******************************
                         FR3                   CDR3
```

Figure S1. Comparison of BAXM159 VH sequence with germline VH subclass 3-23. Software clustal O was used for sequence alignment. * indicates identity, indicates similarity. Frame works, CDR identification and amino acid numbering is indicated according to kabat scheme.

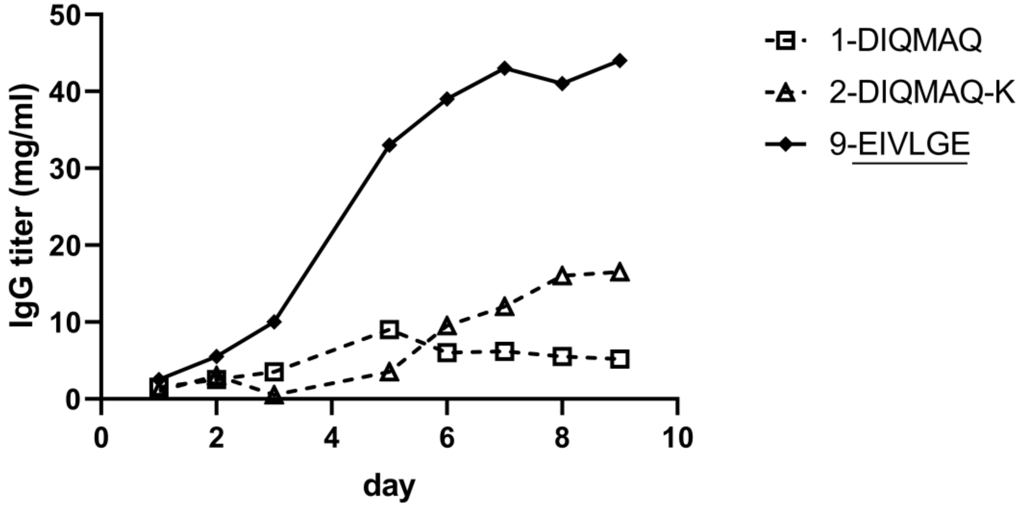

Figure S2. IgG titer of indicated BaxM159 variants at 10 L bioreactor scale. IgG titer was determined by ELISA.

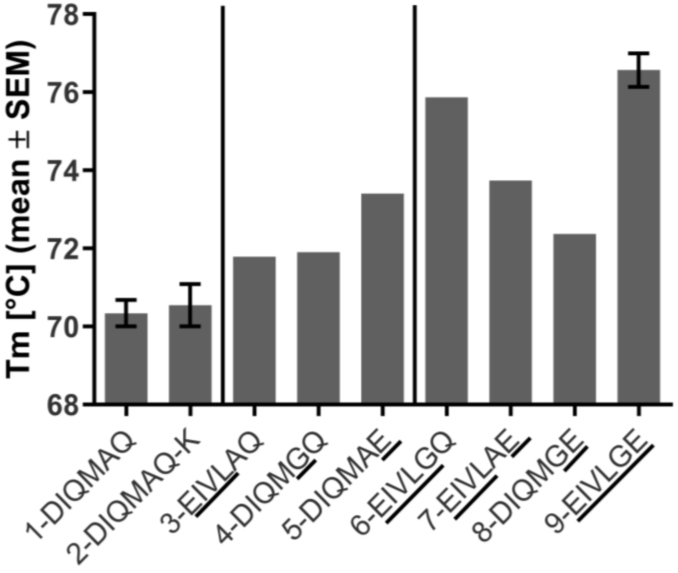

Figure S3. Thermal stability of BaxM159 variants determined by differential scanning calorimetry. Standard error mean (SEM) is indicated by whiskers.

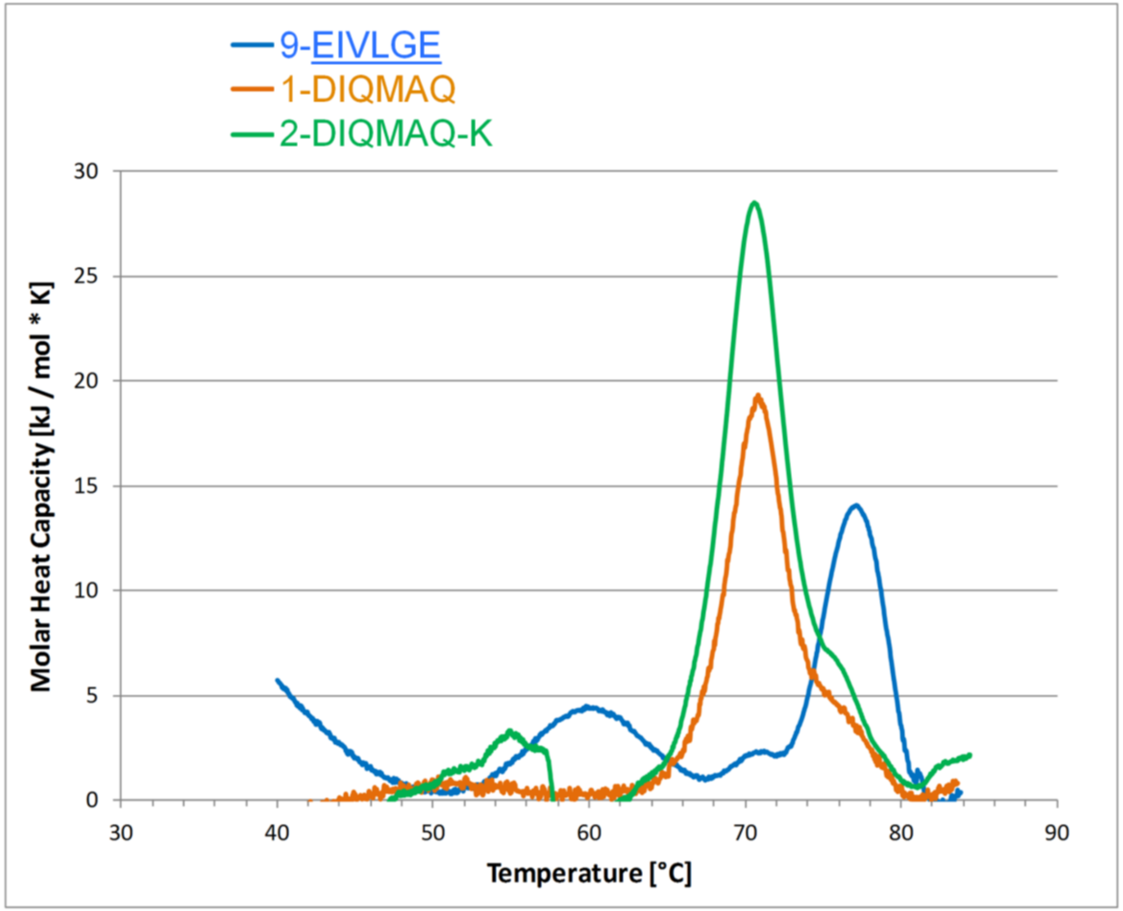

Figure S4. Differential scanning calorimetry thermograms of BaxM159 variants 1-DIQMAQ, 2-DIQMAQ-K and 9-EIVLGE.
